# Neurologic Biomarkers, Neuroimaging, and Cognitive Function in Persistent Atrial Fibrillation: A Cross-Sectional Study

**DOI:** 10.3390/ijms24032902

**Published:** 2023-02-02

**Authors:** Josip Kedžo, Tea Domjanović Škopinić, Josipa Domjanović, Maja Marinović Guić, Sanja Lovrić Kojundžić, Leida Tandara, Andrija Matetić, Zrinka Jurišić

**Affiliations:** 1 Department of Cardiology, University Hospital of Split, 21000 Split, Croatia; 2 Department of Nephrology, University Hospital of Split, 21000 Split, Croatia; 3 Department of Diagnostic and Interventional Radiology, University Hospital of Split, 21000 Split, Croatia; 4 University Department of Health Studies, University of Split, 21000 Split, Croatia; 5 School of Medicine, University of Split, 21000 Split, Croatia; 6 Medical Biochemistry and Laboratory Medicine Subdivision, Medical Laboratory Diagnostic Division, University Hospital of Split, 21000 Split, Croatia

**Keywords:** neurological biomarkers, neuroimaging, magnetic resonance, cognition, atrial fibrillation

## Abstract

The aim of this study was to evaluate the specific neurologic biomarkers, neuroimaging findings, and cognitive function in patients with persistent atrial fibrillation (AF) undergoing electrical cardioversion, compared to control subjects. This cross-sectional study included 25 patients with persistent AF undergoing electrical cardioversion and 16 age- and sex-matched control subjects. Plasma levels of glial fibrillary acidic protein (GFAP), neurofilament light protein (NFL), and ubiquitin carboxyl-terminal hydrolase L1 (UCH-L1), as well as parameters of neuroimaging and cognitive function, were compared between the groups. Neuroimaging was performed using the standard magnetic resonance imaging (MRI) protocol. Cognitive function was assessed using the Patient-Reported Outcomes Measurement Information System (PROMIS) Cognitive Function Index. Further analysis of neurologic biomarkers was performed based on the subsequent electrical cardioversion. There was no significant difference in GFAP (median of 24.7 vs. 28.7 pg/mL, *p* = 0.347), UCH-L1 (median of 112.8 vs. 117.7 pg/mL, *p* = 0.885), and NFL (median of 14.2 vs. 15.4 pg/mL, *p* = 0.886) levels between AF patients and control subjects. Similarly, neuroimaging showed no between-group difference in large cortical and non-cortical lesions (n = 2, 8.0% vs. n = 0, 0.0%, *p* = 0.246), small non-cortical lesions (n = 5, 20.0% vs. n = 5, 31.3%, *p* = 0.413), white matter hyperintensity (n = 23, 92.0% vs. n = 14, 87.5%, *p* = 0.636), and thromboembolic lesions (n = 0, 0.0% vs. n = 1, 6.3%, *p* = 0.206). Cognitive assessment did not show any between-group difference in the PROMIS index (52.2 ± 9.6 vs. 51.2 ± 6.2, *p* = 0.706). Finally, there were no significant dynamics in neurologic biomarkers following electrical cardioversion (*p* > 0.05). This hypothesis-generating study did not find a significant difference in neurologic biomarkers, neuroimaging findings, or cognitive function between patients with persistent AF and controls. The restoration of sinus rhythm was not significantly associated with a change in neurologic biomarkers. Further powered longitudinal studies are needed to re-assess these findings in an AF population.

## 1. Introduction

Atrial fibrillation (AF) is the most prevalent arrhythmia and represents a global health burden. Emerging risk factors, a prolonged life span, and expanded screening have contributed to the increasing incidence of AF, particularly in developed countries [1,2]. Due to the substantial morbidity and mortality, different public health measures have been established to improve the diagnosis and management of these patients [2]. A particular focus has been put on the prevention of acute ischemic stroke, with the CHA2DS2VASc (congestive heart failure, hypertension, age, diabetes mellitus, previous stroke, vascular disease, and sex category) score being the cornerstone of risk stratification and preventative anticoagulation management [2,3].

It has been suggested that AF is associated with neuronal injury and cognitive impairment, irrespective of the previous stroke or co-existing comorbidities [4]. Several contributing mechanisms have been proposed, including genetic factors, cerebral hypoperfusion, systemic inflammation, and silent cerebral ischemia mediated by subclinical microemboli. Furthermore, emerging evidence has demonstrated flow disturbances in AF patients that may disrupt the blood-brain barrier (BBB), thereby exposing the central nervous system to injury with variable degrees of cognitive and psychomotor decline [4,5].

Previous studies have shown that magnetic resonance imaging (MRI) can detect small vascular and non-vascular brain lesions in patients with AF [6]. Even in patients without clinically evident stroke, large cortical or non-cortical infarcts, small non-cortical infarcts, microbleeds, and white-matter hyperintensities have been demonstrated [6,7,8]. Nevertheless, the clinical significance of such isolated findings is questionable, and the importance of comprehensive screening tools that include different neuroimaging, neurocognitive, and laboratory tests has been emphasized.

There is an increasing interest in neurologic biomarkers that could be potentially used for both risk stratification and diagnostic testing in an AF population. Recent studies have suggested that the disruption of the BBB could induce the peripheral release of markers of neuronal injury, such as glial fibrillary acidic protein (GFAP) and serum neurofilament light protein (NFL) [9,10,11]. Ubiquitin carboxyl-terminal hydrolase L1 (UCH-L1) is an additional emerging biomarker that is specific to the central nervous system but can be found in the peripheral circulation after a traumatic brain injury [12]. Although there are individual studies investigating GFAP and NFL, there are no comprehensive data evaluating all aforementioned neurologic biomarkers in an AF population, including their association with electrical cardioversion.

Therefore, the goal of this study was to determine plasma levels of specific neurologic biomarkers (GFAP, NFL, and UCH-L1), neuroimaging findings, and cognitive function in patients with persistent AF undergoing electrical cardioversion and compare them to those in control patients. Furthermore, this study investigated the dynamics of these biomarkers regarding electrical cardioversion in AF patients.

## 2. Results

There was no statistically significant difference in the baseline characteristics between the AF patients and the control group, except in heart rate and values of NTproBNP that were higher in AF patients (median of 90 vs. 68 bpm, *p* < 0.001, and median of 895.0 vs. 85.0 pg/mL, respectively) (Table 1). Patients with AF were adequately anticoagulated (median of 10.5 weeks), with preserved left ventricular ejection fraction (median of 59.0%) and increased left atrial diameter (median of 48.0 mm). Most patients had long-lasting persistent AF (median of 67.0 days) (Supplementary Table S1) and were symptomatic with an EHRA class >1 in 96.0% of patients (Supplementary Figure S2).

### 2.1. Specific Neurologic Biomarkers

**Figure 1:**
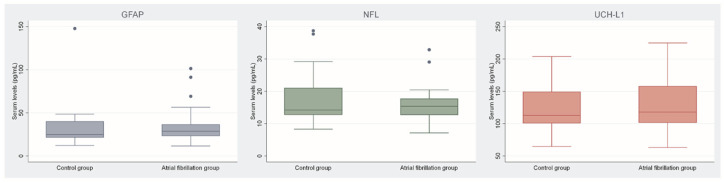
Comparison of biomarkers between the atrial fibrillation group and the control group. Legend: GFAP—glial fibrillary acidic protein; NFL—neurofilament light chain; UCH-L1—ubiquitin C-terminal hydrolase L1.

When comparing the neurological biomarkers between the AF patients and the control group, there was no significant difference in GFAP (median of 24.7 vs. 28.7 pg/mL, *p* = 0.347), UCH-L1 (median of 112.8 vs. 117.7 pg/mL, *p* = 0.885), and NFL (median of 14.2 vs. 15.4 pg/mL, *p* = 0.886) (Table 1 and Figure 1).

**Figure 2:**
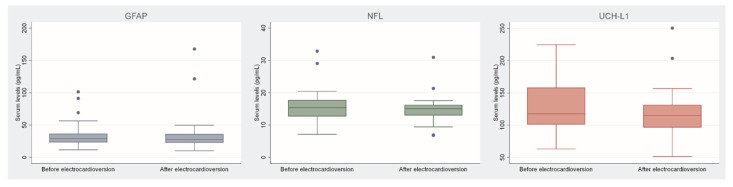
Comparison of biomarkers within the atrial fibrillation group based on the electrical cardioversion. Legend: GFAP—glial fibrillary acidic protein; NFL—neurofilament light chain; UCH-L1—ubiquitin C-terminal hydrolase L1.

Neurological biomarkers within the AF group did not differ significantly before and after electrical cardioversion for GFAP (median of 28.7 pg/mL before electrical cardioversion vs. 27.7 pg/mL after electrical cardioversion, *p* = 0.347), UCH-L1 (median of 115.8 pg/mL before electrical cardioversion vs. 114.9 pg/mL after electrical cardioversion, *p* = 0.885), and NFL (median of 15.4 pg/mL before electrical cardioversion vs. 15.0 pg/mL after electrical cardioversion, *p* = 0.886) before and after electrical cardioversion (Figure 2).

### 2.2. Neuroimaging with Magnetic Resonance

Neuroimaging with magnetic resonance revealed no difference between the AF patients and the control group in large cortical and non-cortical lesions (n = 2, 8.0% vs. n = 0, 0.0%, *p* = 0.246), small non-cortical lesions (n = 5, 20.0% vs. n = 5, 31.3%, *p* = 0.413), white matter hyperintensity (n = 23, 92.0% vs. n = 14, 87.5%, *p* = 0.636), and acute or subacute thromboembolic lesions (n = 0, 0.0% vs. n = 1, 6.3%, *p* = 0.206) (Table 2). Consistently, there was no significant difference in the Fazekas scale between the AF patients and the control group (1.2 ± 0.8 vs. 1.1 ± 0.8, *p* = 0.775). Interestingly, the control subjects had a statistically significantly higher occurrence of asymptomatic cerebral microbleeding on magnetic resonance imaging (n = 3, 18.8% vs. n = 0, 0.0%, *p* = 0.025) (Table 2).

### 2.3. Cognitive Function Assessment

Cognitive assessment did not show any difference between the AF patients and the control group in the PROMIS index (52.2 ± 9.6 vs. 51.2 ± 6.2, *p* = 0.706), nor in any of its dimensions (*p* > 0.05) (Table 3).

### 2.4. Correlation Analysis

The correlation analysis revealed a significant association between GFAP and UCH-L1 (r = 0.383, *p* = 0.028), age (r = 0.369, *p* = 0.035), Hgb (r = −0.556, *p* < 0.001), NTproBNP (r = 0.465, *p* = 0.006), and vWF (r = 0.381, *p* = 0.029), as well as between the UCH-L1 and eGFR (r = −0.365, *p* = 0.037) and vWF (r = 0.362, *p* = 0.039), while there was no significant association of NFL and selected variables (*p* > 0.05) (Supplementary Table S2).

## 3. Discussion

This is the first study that comprehensively evaluated the levels of selected neurologic biomarkers, magnetic resonance neuroimaging, and cognitive function in patients with persistent AF, compared to age- and sex-matched control subjects. Whether neurologic biomarkers have an additional screening role in this population on top of MRI and cognitive function has not been well reported. In addition, potentially useful neurologic biomarkers such as UCH-L1 were not previously investigated in AF patients, while the assessment of neurologic biomarkers with regard to electrical cardioversion includes an additional novelty to the literature. There are several important findings in this study. First, patients with AF did not exhibit higher levels of GFAP, NFL, or UCH-L1 compared to matched control subjects. Second, restoration of sinus rhythm with electrical cardioversion was not associated with a change in levels of GFAP, NFL, and UCH-L1 amongst patients with persistent AF. Third, there was a significant positive correlation between GFAP and UCH-L1, while there was no association with NFL. Fourth, both GFAP and UCH-L1 exhibit significant association with increasing vWF, while GFAP showed additional association with increasing age, lower hemoglobin, and increasing NTproBNP. Finally, AF patients did not exhibit significantly different findings in magnetic resonance neuroimaging and cognitive assessment compared to the control subjects.

Co-existing neuronal injury in patients with AF may manifest as cognitive or psychomotor impairment. The present study did not reveal statistically significant differences in cognitive function or selected neurologic biomarkers between the AF patients and the control subjects. A previous study by Galenko et al. reported a statistically significant difference between the abovementioned groups, with higher values of GFAP in the AF patients. However, this study did not use an MRI of the brain, and it is possible that some patients had silent cerebral lesions that could have affected the results. The same study also detected a significant association of GFAP with older age and a higher CHA2DS2VASc score [9]. Elevated levels of GFAP could indirectly indicate BBB disruption and early neuronal injury that may be important in an AF population, considering their substantial cognitive and psychomotor burden. A significant association between GFAP and older age was confirmed in our study, but there was no correlation with the CHA2DS2VASc risk score. Further, the present study did not reveal a between group-difference in NFL levels, which is in contrast to previous studies that showed higher NFL values in an AF population [10,11]. However, a study by Polymeris et al. did not have a control group of patients, and the exclusion criterion was only recent (< 4 weeks) stroke which may have influenced the study population [10]. Furthermore, MRI was not routinely utilized in a study by Sjölin et al., which represents an important limitation [11]. Previous studies have shown a significant association between NFL and age, diabetes mellitus, renal dysfunction, blood pressure, heart failure, and cerebrovascular lesions [10,13]. The present study did not detect an association between NFL levels and the aforementioned factors. Importantly, to the best of our knowledge, there have been no studies investigating UCH-L1 in an AF population, even though this study did not show a significant difference in UCH-L1 levels between the study groups. Considering the limited sample size of the present study, further research is necessary to clarify the role of UCH-L1 in an AF population.

When accounting for the electrical cardioversion, there are no studies that examined the levels of these neurologic biomarkers before and after conversion to stable sinus rhythm. It has been previously suggested that AF could potentially induce BBB damage, and this study hypothesized that restoration of a sinus rhythm could be associated with a reduction of neurologic biomarkers. A study by Sjölin et al. evaluated NFL levels in patients with a history of AF, stratified by heart rhythm at the time of blood sampling (sinus rhythm vs. AF), but without MRI data. The authors report lower levels of NFL in patients with sinus rhythm compared to those with persistent AF [11]. We did not find a significant difference before and 6 weeks after the electrical cardioversion. There are several possible explanations for these results. First, the burden of AF may have a crucial role in the neurological injury, with long-standing AF having a worse impact compared to AF of a shorter duration. Second, it is unclear if any of the AF patients developed subclinical paroxysmal episodes during the 6 week period after the electrical cardioversion, within the sampling interval period. Finally, the limited sample size could affect the statistical strength of the study. Further focused research is encouraged to clarify these hypothesis-generating findings.

The present study showed no difference in MRI findings between the AF patients and the control subjects, including the number of cortical and non-cortical brain lesions. This is in contrast with a previous study that reported a higher risk of subclinical brain infarctions in patients with AF. However, this study represents data from the old era prior to direct oral anticoagulants [14]. The present study did not show a significant difference in white matter hyperintensities. One of the possible explanations can be found in the characteristics of control subjects. Specifically, the control group in this study represents subjects without AF but who had other important comorbidities and cardiovascular risk factors (such as diabetes, hypertension, or smoking) that could per se induce brain lesions. The available literature data on this topic is inconsistent. A recent study also showed no association between AF and white matter hyperintensities [15], while Kobayashi et al. proved that AF is an independent risk factor for white matter hyperintensities [16]. In addition, this study showed no difference in cognitive function as assessed by the PROMIS index. Although available evidence suggests important neurocognitive impairments in AF patients [17,18], there are no available studies using the PROMIS index. Furthermore, this study includes a simultaneous evaluation of cognitive function, MRI neuroimaging findings, and selected neurologic biomarkers that could have a complementary role in an AF population.

There are several limitations to this study. First, the limited sample size could affect the statistical power of the study. A limited sample size prevents further sensitivity analyses, such as the analysis by NT-proBNP level categories, as this would decrease the statistical strength and increase the possibility of a type 1 error. Second, the data were collected from a single center, leading to potential selection and treatment biases. Third, there is no standardized time frame between electrical cardioversion and repeated measurement of neurologic biomarkers due to a lack of literature evidence. It remains unclear whether a 6 week interval was adequate for the analysis of biomarkers and whether there were subclinical events such as subclinical AF episodes in this period. Due to the limited sample size of this study, further longitudinal and powered studies are warranted, while the clinical applicability of the study results is dependent on their validation in a larger sample of patients. Although this study did not reveal significant differences between the study groups, the increasing incidence of AF warrants objective screening tools that could timely detect potential brain injury in this prevalent population.

In conclusion, this study did not find any significant differences in specific neurologic biomarkers, neuroimaging findings, or cognitive function between patients with persistent AF and the control subjects. The restoration of a sinus rhythm with electrical cardioversion was not associated with a significant change in plasma levels of specific neurologic biomarkers. Further powered longitudinal studies are needed to investigate the role of these biomarkers, on top of MRI and cognitive tests, in an AF population.

## 4. Materials and Methods

### 4.1. Ethical Considerations and Informed Consent

The study was conducted according to the Helsinki Declaration and approved by the Medical Research Ethical Committee of the University Hospital of Split (No. 2181-147/01/06/M.S.-21-02). All included subjects have provided informed written consent prior to the study’s initiation. All the procedures within the study have been conducted in line with the principles of good clinical practice.

### 4.2. Study Design and Patients

This single-center cross-sectional study was conducted at the University Hospital of Split and enrolled a total of 41 subjects, including 25 subjects with persistent atrial fibrillation undergoing elective electrical cardioversion and 16 age- and sex-matched control subjects with a similar comorbidity burden. All AF patients were anticoagulated for at least 3 weeks prior to the electrical cardioversion, and transoesophageal echocardiography was not done prior to the electrical cardioversion. All enrolled subjects conformed to the study protocol, which included the analysis of selected neurologic biomarkers (GFAP, NFL, and UCH-L1), neuroimaging, and cognitive assessment. Exclusion criteria were: prior known cerebrovascular event (ischemic stroke, transient ischemic attack, or haemorrhagic stroke), long-standing persistent AF (>1 year duration of the ongoing episode); left ventricular ejection fraction <40%; severe valvular disease; diagnosis of significant (>70%) stenosis of carotid arteries; prior intervention on carotid arteries; any diagnosed neurologic disease, any diagnosed psychiatric disorder, history of malignant disease, and intolerance of anticoagulants. The flow diagram of the study is shown in Supplementary Figure S1. The study was reported according to the Strengthening the Reporting of Observational Studies in Epidemiology (STROBE) guidelines.

### 4.3. Outcomes

The primary outcome of this study was the comparison of plasma levels of neurologic biomarkers (GFAP, NFL, and UCH-L1) between patients with persistent atrial fibrillation and control subjects. Secondary outcomes included a qualitative and quantitative comparison of neuroimaging with magnetic resonance between patients with persistent AF and control subjects and a comparison of cognitive function between patients with persistent AF and control subjects. Additional analysis was conducted to compare the plasma levels of neurological biomarkers (GFAP, NFL, and UCH-L1) within patients with persistent AF, before and 6 weeks after the electrical cardioversion.

### 4.4. Laboratory Analysis and Specific Neurologic Biomarkers

All study participants underwent laboratory analysis that included these parameters: low-density lipoprotein cholesterol (LDL-C), creatinine, haematocrit level (Hct), hemoglobin level (Hgb), N-terminal pro-brain natriuretic peptide (NTproBNP), high-sensitivity troponin T (hsTnT), von Willenbrand factor (vWF), albumin, and glucose. The estimated glomerular filtration rate (eGFR) was determined using the CKD-EPI (Chronic Kidney Disease Epidemiology Collaboration) equation.

GFAP is a neurologic biomarker that is primarily present in astroglial cells of the central nervous system. It is largely specific for the central nervous system, although it can be found in Schwann cells of the peripheral nervous system and enteric glial cells [19]. Its levels are very low in the peripheral blood of healthy individuals under these physiological circumstances [20]. However, it has been previously shown that increased plasma levels of GFAP can be seen in cases of traumatic brain or spinal cord injury and stroke [21]. The atomic mass of GFAP is ~50 kDa with a plasma half-life of 24–48 h [22].

UCH-L1 is part of the ubiquitin-proteasome system that is expressed primarily in neuroendocrine cells and the central nervous system and has a complex role in the regulation of protein degradation. It is sometimes found in tumors originating from tissues that usually do not express it, like pancreatic, breast, and colorectal malignancies. In the central nervous system, UCH-L1 has an important role in the repair of damaged neurons and axons [12]. Serum levels are increased in cases of traumatic brain injury. The atomic mass of UCH-L1 is ~26 kDa with a plasma half-life of 7–9 h [22,23]. Its relevance to AF patients has been based on a blood-brain barrier injury during arrhythmia, although there has not been any available data regarding the levels of UCH-L1 in patients with AF.

Serum neurofilament light proteins (NFL) are axonal proteins that are found in large, myelinated axons of the central and peripheral nervous systems and are released into the circulation in cases of neuroaxonal damage [24]. Their levels have been shown to be increased in different neurological conditions, such as ischemic stroke and multiple sclerosis [25]. The atomic mass of NFL is ~70 kDa, while its plasma half-life varies from several days to several weeks [22,24].

### 4.5. Neuroimaging by Magnetic Resonance

Magnetic resonance imaging analysis was conducted on all subjects. It was performed using a 1.5 Tesla scanner (Magnetom Aera; Siemens Healthcare GmbH, 91052 Erlangen, Germany) with the 16-channel acquisition coil. The patients were placed in the supine position, and the head was safely placed in the head coil for fixation to avoid motion artifacts.

The standardized protocol included the following sequences: three-dimensional flash T1, 3D fluid-attenuation inversion recovery (FLAIR), coronal T2-weighted, diffusion-weighted image (DWI), and susceptibility-weighted image (SWI). The DWI (b: 1000 s/mm^2^) protocol was performed using the following parameters: a repetition time of 4000 ms; an echo time of 86 ms; and a slice thickness of 5 mm. Automatically generated apparent diffusion coefficient (ADC) maps were subsequently reviewed.

Two board-certified neuroradiologists (S.L.K. and M.M.G.) blinded to patient characteristics, electrical cardioversion procedures, or laboratory findings evaluated the MRI scans. According to the adaptation of the standards for reporting vascular changes on neuroimaging [8,10], we analyzed the following brain lesions on the 3D FLAIR sequence: small noncortical infarcts (SNCIs), large noncortical infarcts (LNCIs), cortical infarcts, hyperintense white matter lesions (WMLs), and microbleeds (MBs). SNCIs included lesions that were hyperintense on FLAIR sequence without involving the cortex because of their location in the territory of the perforating arteriole. LNCIs were defined as infarcts larger than 20 mm and sparing the cortex. Cortical infarcts include lesions involving the cortex. Large noncortical and cortical infarcts were analyzed as one group (LNCCIs). The WMLs are those lesions that do not meet the previously mentioned criteria for infarctions and are graded according to the Fazekas scale [26]. MBs were defined by susceptibility-weighted imaging as punctate or nodular hyperintensities on phase images. Acute ischemic lesions were identified as DWI hyperintensities with the corresponding hypointensities on ADC maps.

All study subjects underwent baseline magnetic resonance imaging, while the AF patients underwent additional magnetic resonance imaging (6 weeks after the electrical cardioversion).

### 4.6. Cognitive Assessment

A cognitive assessment was performed using the self-reported Patient-Reported Outcomes Measurement Information System (PROMIS) Cognitive Function Index, which consists of an eight item short form, as previously described [27].

### 4.7. Clinical Assessment

A detailed clinical assessment was performed on all subjects. Arterial blood pressure was measured using the sphygmomanometer in the sitting position after at least 10 minutes of rest. The mean arterial pressure (MAP) was then determined as the sum of 2/3 of the diastolic blood pressure and 1/3 of the systolic blood pressure. Body mass index (BMI) was calculated as a division of body mass (kg) and squared body height (m^2^). The CHA_2_DS_2_VASc and HAS-BLED (Hypertension, Abnormal Renal/Liver Function, Previous Stroke, History or Predisposition to Bleeding, Labile INR, Age, Concomitant Use of Drugs/Alcohol) risk scores were calculated according to the previous recommendations [3,28]. Heart rate was determined by a 12-lead electrocardiogram prior to electrical cardioversion.

### 4.8. Statistical Methods

Statistical analysis was performed using Stata software (StataCorp, College Station, TX, USA; version 17). To account for the non-normal data distribution, we have primarily used non-parametric tests and reported results as the median (interquartile range). Only the data for cognitive function were reported as mean ± standard deviation due to a parametric distribution. Categorical data were described as numbers (percentages). The Chi-squared (χ^2^) test was used for the comparison of categorical data, while continuous data were compared by using the Mann–Whitney U test and t-test, respectively. Spearman’s rank correlation coefficient analysis was used to determine the association of neurological biomarkers with selected quantitative parameters, including age, BMI, MAP, LDL, eGFR, Hgb, NTproBNP, hsTnT, vWF, albumin, glucose, CHA_2_DS_2_VASc score, and HAS-BLED score. It was expressed as Spearman’s rank correlation coefficient (rs) and its associated *p*-values. Statistical significance was set at the level of *p* < 0.05.

## Figures and Tables

**Table 1:** Comparison of baseline characteristics between the study groups.

Variables	Control Group (n = 16; 39.0%)	Atrial Fibrillation Group (n = 25; 61.0%)	*p*-Value
Age (years)	67 (63, 69)	68 (63, 72)	0.768 *
Female sex	6 (37.5%)	9 (36.0%)	0.923 *
Body mass index (kg/m^2^)	28 (26, 31)	29 (26, 31)	0.490 †
Systolic blood pressure (mmHg)	132 (122, 152)	137 (129, 144)	0.769 †
Diastolic blood pressure (mmHg)	78 (74, 88)	80 (73, 82)	0.928 †
Mean blood pressure (mmHg)	95 (93, 109)	100 (91, 102)	0.916 †
Heart rate (/min)	68 (58, 73)	90 (78, 96)	<0.001 †
CHA_2_DS_2_VASc risk score	2 (1, 3)	2 (2, 3)	0.506 †
HAS-BLED risk score	1 (0, 1)	1 (0, 1)	0.965 †
Laboratory parameters			
Total cholesterol (mmol/L)	5.5 (4.7, 6.2)	5.5 (4.1, 5.8)	0.228 †
LDL cholesterol (mmol/L)	3.4 (2.5, 3.7)	3.3 (2.3, 3.8)	0.591 †
Creatinine (µmol/L)	79.0 (75.0, 87.0)	83.5 (74.8, 101.8)	0.271 †
eGFR (ml/min/1.73 m^2^)	80.0 (67.0, 87.0)	67.5 (52.0, 90.3)	0.500 †
Hematocrite (%)	0.42 (0.41, 0.43)	0.43 (0.39, 0.46)	0.702 †
Hemoglobin (g/L)	142.0 (141.0, 155.0)	142.5 (131.3, 154.8)	0.344 †
NT-proBNP (pg/mL)	85.0 (53.0, 157.0)	895.0 (678.0, 1430.8)	<0.001 †
hsTnT (ng/mL)	11.6 (9.3, 14.4)	10.2 (6.8, 12.3)	0.109 †
Glucose (mmol/L)	5.5 (5.3, 7.6)	5.9 (5.2, 6.8)	0.295 †
Albumine (g/L)	43.3 (41.3, 46.8)	44.2 (42.1, 47.0)	0.682 †
vWF	1.3 (1.1, 1.7)	1.7 (1.4, 1.7)	0.038 †
Comorbidities			
Arterial hypertension	12 (75.0%)	18 (72.0%)	0.833 *
Diabetes mellitus	3 (18.8%)	4 (16.0%)	0.819 *
Chronic kidney disease	0 (0.0%)	(0.0%)	/
Smoking:			0.127 *
Active smoking	4 (25.0%)	7 (28.0%)	
Prior smoking	7 (43.8%)	4 (16.0%)	
Medications			
Beta blockers	3 (18.8%)	22 (88.0%)	<0.001 *
Statins	4 (25.0%)	7 (28.0%)	0.833 *
ASA	4 (25.0%)	/	/
Use of anticoagulation:			<0.001 *
Warfarin	0 (0.0%)	3 (12.0%)	
DOAC	1 (6.3%)	22 (88.0%)	
Biomarkers			
GFAP (pg/mL)	24.7 (21.3, 42.3)	28.7 (22.9, 38.6)	0.347 †
UCH-L1 (pg/mL)	112.8 (99.6, 152.0)	117.7 (100.9, 159.8)	0.885 †
NFL (pg/mL)	14.2 (12.7, 21.5)	15.4 (12.3, 17.7)	0.886 †

Data are expressed as a number (percentage) or median (interquartile range). * Chi-square test; † Mann–Whitney U test. Note: Heart rate was determined by a 12-lead electrocardiogram prior to electrical cardioversion. Legend: ASA—acetylsalicylic acid; DOAC—direct oral anticoagulants; eGFR—estimated glomerular filtration rate; GFAP—glial fibrillary acidic protein; hsTnT—high-sensitive troponin T; LDL—low-density lipoprotein; NFL—neurofilament light chain; NTproBNP—N-terminal pro-brain natriuretic peptide; UCH-L1—ubiquitin C-terminal hydrolase L1; vWF—von Willebrand factor.

**Table 2:** Comparison of cerebrovascular findings on magnetic resonance imaging between the study groups.

Variables	Control Group (n = 16; 39.0%)	Atrial Fibrillation Group (n = 25; 61.0%)	*p*-Value
Magnetic resonance imaging			
Large cortical and non-cortical lesions	0 (0.0%)	2 (8.0%)	0.246 *
Large cortical and non-cortical lesions (number)	/	0.1 ± 0.3	/
Small non-cortical lesions	5 (31.3%)	5 (20.0%)	0.413 *
Small non-cortical lesions (number)	1.2 ± 3.0	0.6 ± 1.4	0.373 †
Microbleeding	3 (18.8%)	0 (0.0%)	0.025 *
Microbleeding (number)	1.4 ± 5.0	/	0.172 †
White matter hyperintensity	14 (87.5%)	23 (92.0%)	0.636 *
Acute or subacute thromboembolic lesions	1 (6.3%)	0 (0.0%)	0.206 *
Fazekas scale	1.1 ± 0.8	1.2 ± 0.8	0.775 †

Data are expressed as a number (percentage) or mean ± standard deviation. * Chi-square test; † *t*-test. Legend: None.

**Table 3:** Comparison of cognitive function scores between the study groups.

Variables	Control Group (n = 16; 39.0%)	Atrial Fibrillation Group (n = 25; 61.0%)	*p*-Value *
PROMIS index	51.2 ± 6.2	52.2 ± 9.6	0.706
PROMIS dimension 1: Slower thinking	3.8 ± 0.8	4.2 ± 1.0	0.205
PROMIS dimension 2: Impression of brain thinking impairment	4.3 ± 0.8	4.2 ± 0.9	0.595
PROMIS dimension 3: Need for a stronger focus on everyday activities	4.6 ± 0.6	4.3 ± 0.9	0.295
PROMIS dimension 4: Impairment in multi-tasking	4.4 ± 0.8	4.3 ± 1.1	0.762
PROMIS dimension 5: Concentration impairment	4.1 ± 0.9	4.0 ± 1.1	0.796
PROMIS dimension 6: Need for stronger focus to avoid mistakes	4.2 ± 0.9	4.2 ± 1.0	0.967
PROMIS dimension 7: Impairment in idea shaping	4.5 ± 0.5	4.1 ± 1.1	0.215
PROMIS dimension 8: Impairment in number calculation	4.6 ± 0.6	4.5 ± 0.9	0.872

Data are expressed as mean ± standard deviation. * *t*-test.

## Data Availability

The data used during this study are available on reasonable request from the corresponding author.

## Supplementary Materials

The following supporting information can be downloaded at: https://www.mdpi.com/article/10.3390/ijms24032902/s1.
