# Supplementary material for: Neurologic Biomarkers, Neuroimaging, and Cognitive Function in Persistent Atrial Fibrillation: A Cross-Sectional Study
Source: Int J Mol Sci. 2023 Feb 2;24(3):2902. doi: 10.3390/ijms24032902 (PMC9918133; doi:10.3390/ijms24032902)
Supplement: Supplementary file 1 [file ijms-24-02902-s001.zip › ijms-2193322-supplementary.pdf]

SUPPLEMENTARY TABLES AND FIGURES

Supplementary Table S1. Atrial fibrillation-related factors in the study cohort

Variables	Atrial fibrillation group (n=25; 61.0%)
**Duration of anticoagulation (weeks)**	10.5 (7.3, 19.8)
**Duration of atrial fibrillation (days)**	67.0 (45.5, 105.8)
**1st episode of atrial fibillation**	18 (72.0%)
**Left atrial diameter (mm)**	48.0 (43.0, 51.0)
**Ejection fraction (%)**	59.0 (50.0, 66.5)
**Prior electro cardioversion**	7 (28.0%)
**Prior pulmonary vein isolation**	1 (4.0%)
**Successful electrical cardioversion**	22 (88.0%)
**Atrial fibrillation recurrence**	9 (36.0%)
**Antiarrhythmics**	9 (36.0%)

Data are expressed as number (percentage) or median (interquartile range).
Abbreviations: None.

Supplementary Table S2. Correlation between biomarkers and selected variables.

Variables	GFAP	UCH-L1	NFL	Age	BMI	MAP	LDL	eGFR	Hgb	NTproBNP	hsTnT	vWF	Albumin	Glucose	CHA_2DS_2VASc	HAS-BLED
	rs (p-value)															
**GFAP**		0.383 (0.028)	0.309 (0.080)	0.369 (0.035)	0.214 (0.232)	0.043 (0.812)	-0.071 (0.695)	-0.300 (0.090)	-0.556 (<0.001)	0.465 (0.006)	-0.223 (0.212)	0.381 (0.029)	-0.303 (0.087)	-0.138 (0.445)	0.269 (0.131)	0.202 (0.260)
**UCH-L1**			0.033 (0.856)	0.267 (0.133)	0.324 (0.066)	-0.228 (0.202)	-0.236 (0.186)	-0.365 (0.037)	-0.093 (0.607)	0.245 (0.169)	0.153 (0.396)	0.362 (0.039)	-0.240 (0.179)	0.033 (0.854)	0.320 (0.069)	0.242 (0.175)
**NFL**				-0.055 (0.760)	-0.086 (0.635)	0.045 (0.806)	-0.222 (0.214)	0.150 (0.406)	0.093 (0.605)	0.020 (0.910)	0.120 (0.505)	0.224 (0.210)	0.038 (0.832)	0.114 (0.528)	0.141 (0.433)	0.225 (0.207)
**Age**					0.374 (0.032)	0.341 (0.052)	0.174 (0.332)	-0.329 (0.062)	-0.225 (0.207)	0.267 (0.133)	0.117 (0.518)	0.066 (0.718)	-0.055 (0.762)	-0.174 (0.332)	0.454 (0.008)	0.652 (<0.001)
**BMI**						0.480 (0.005)	0.154 (0.393)	-0.252 (0.156)	-0.163 (0.365)	0.115 (0.523)	0.151 (0.403)	0.160 (0.375)	0.054 (0.765)	-0.048 (0.793)	0.197 (0.272)	0.115 (0.523)
**MAP**							0.230 (0.197)	-0.123 (0.496)	-0.082 (0.652)	0.028 (0.877)	0.053 (0.769)	0.071 (0.696)	0.273 (0.124)	-0.084 (0.641)	0.211 (0.238)	0.210 (0.240)
**LDL**								-0.122 (0.498)	-0.323 (0.066)	0.022 (0.905)	-0.294 (0.096)	-0.126 (0.487)	0.190 (0.290)	-0.301 (0.088)	-0.090 (0.618)	0.014 (0.937)
**eGFR**									0.354 (0.043)	-0.316 (0.074)	0.275 (0.122)	0.106 (0.557)	-0.147 (0.414)	0.116 (0.520)	-0.375 (0.031)	-0.055 (0.761)
**Hgb**										-0.498 (0.003)	0.235 (0.189)	-0.230 (0.197)	0.275 (0.121)	0.298 (0.092)	-0.254 (0.154)	0.586 (0.746)
**NTproBNP**											-0.189 (0.291)	0.519 (0.002)	-0.093 (0.608)	-0.017 (0.927)	0.250 (0.161)	0.157 (0.383)
**hsTnT**												0.139 (0.442)	-0.048 (0.790)	0.447 (0.009)	0.296 (0.095)	0.278 (0.117)
**vWF**													-0.305 (0.085)	0.290 (0.101)	0.239 (0.180)	0.197 (0.272)
**Albumin**														0.188 (0.295)	-0.135 (0.455)	-0.075 (0.678)
**Glucose**															0.093 (0.606)	-0.097 (0.592)

**CHA_2DS_2VASc**	0.667 (<0.001)
**HAS-BLED**	

rs – Spearman's rho correlation coefficient.

Legend: eGFR – estimated glomerular filtration rate; GFAP – glial fibrillary acidic protein; Hgb – hemoglobin; hsTnT – high-sensitive troponin T; LDL – low-density lipoprotein; NFL – neurofilament light chain; NTproBNP – N-terminal pro-brain natriuretic peptide; UCH-L1 – ubiquitin C-terminal hydrolase L1; vWF – von Willebrand factor.

Supplementary Figure S1. Flow diagram

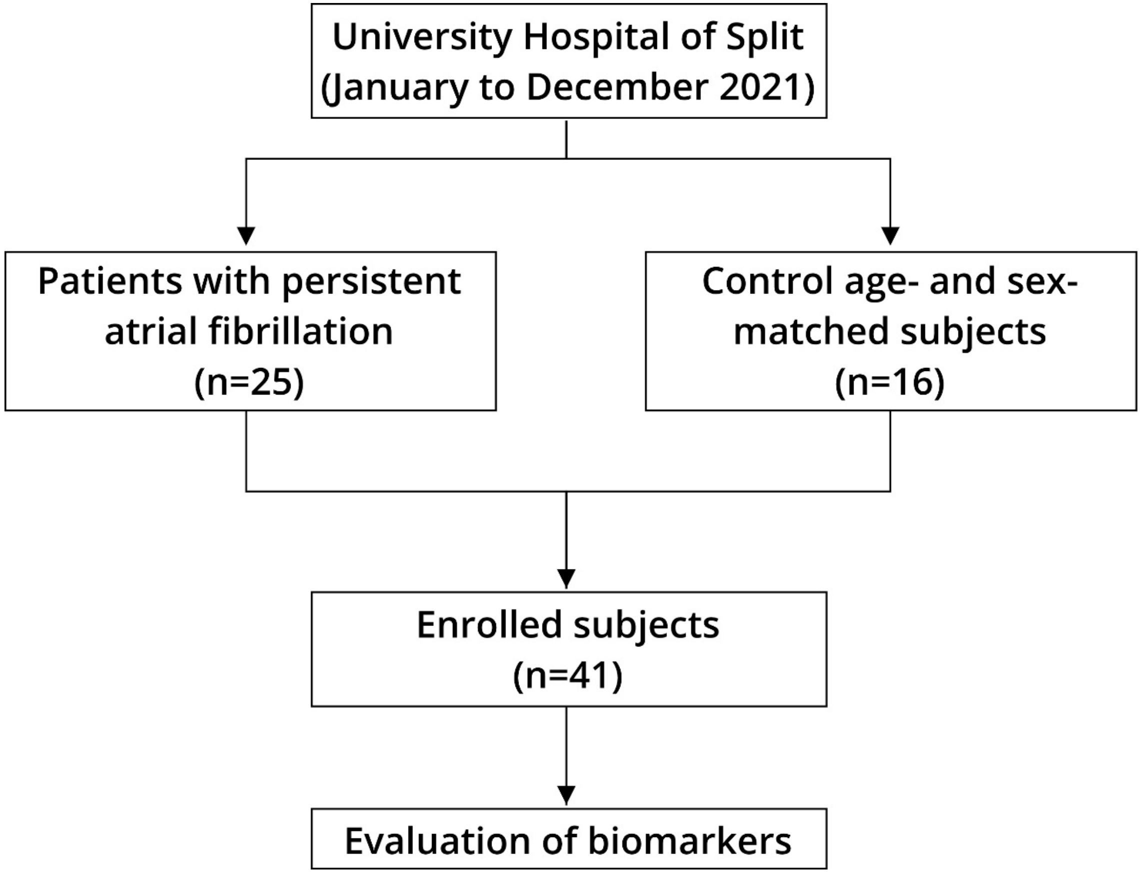

Abbreviations: none.

Supplementary Figure S2. Distribution of EHRA class in the atrial fibrillation group.

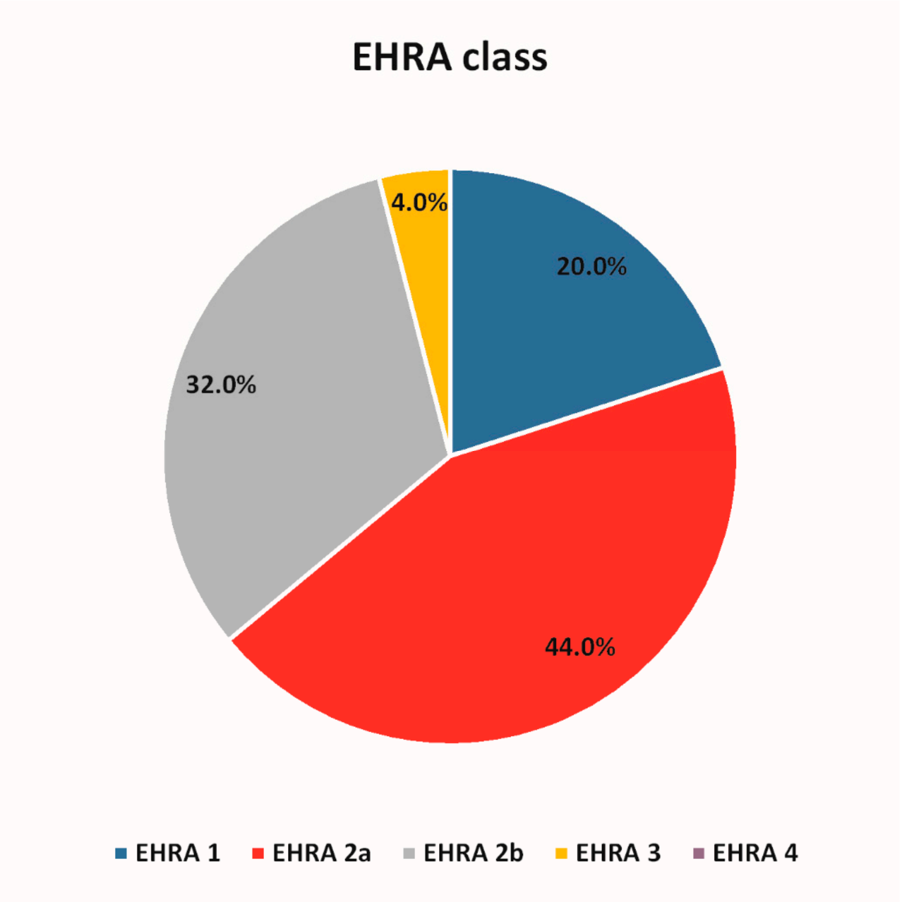

Legend: European Heart Rhythm Association classification.
